# Interfacing medicinal chemistry with structural bioinformatics: implications for T box riboswitch RNA drug discovery

**DOI:** 10.1186/1471-2105-13-S2-S5

**Published:** 2012-03-13

**Authors:** Franziska Jentzsch, Jennifer V Hines

**Affiliations:** 1 Department of Chemistry, Leipzig University, Leipzig, Germany; 2 Department of Chemistry & Biochemistry, Ohio University, Athens, OH, USA

## Abstract

**Background:**

The T box riboswitch controls bacterial transcription by structurally responding to tRNA aminoacylation charging ratios. Knowledge of the thermodynamic stability difference between two competing structural elements within the riboswitch, the terminator and the antiterminator, is critical for effective T box-targeted drug discovery.

**Methods:**

The ΔG of aminoacyl tRNA synthetase (aaRS) T box riboswitch terminators and antiterminators was predicted using DINAMelt and the resulting ΔΔG (ΔG_Terminator _- ΔG_Antiterminator_) values were compared.

**Results:**

Average ΔΔG values did not differ significantly between the bacterial species analyzed, but there were significant differences based on the type of aaRS.

**Conclusions:**

The data indicate that, of the bacteria studied, there is little potential for drug targeting based on overall bacteria-specific thermodynamic differences of the T box antiterminator vs. terminator stability, but that aaRS-specific thermodynamic differences could possibly be exploited for designing drug specificity.

## Background

**Figure 1:**
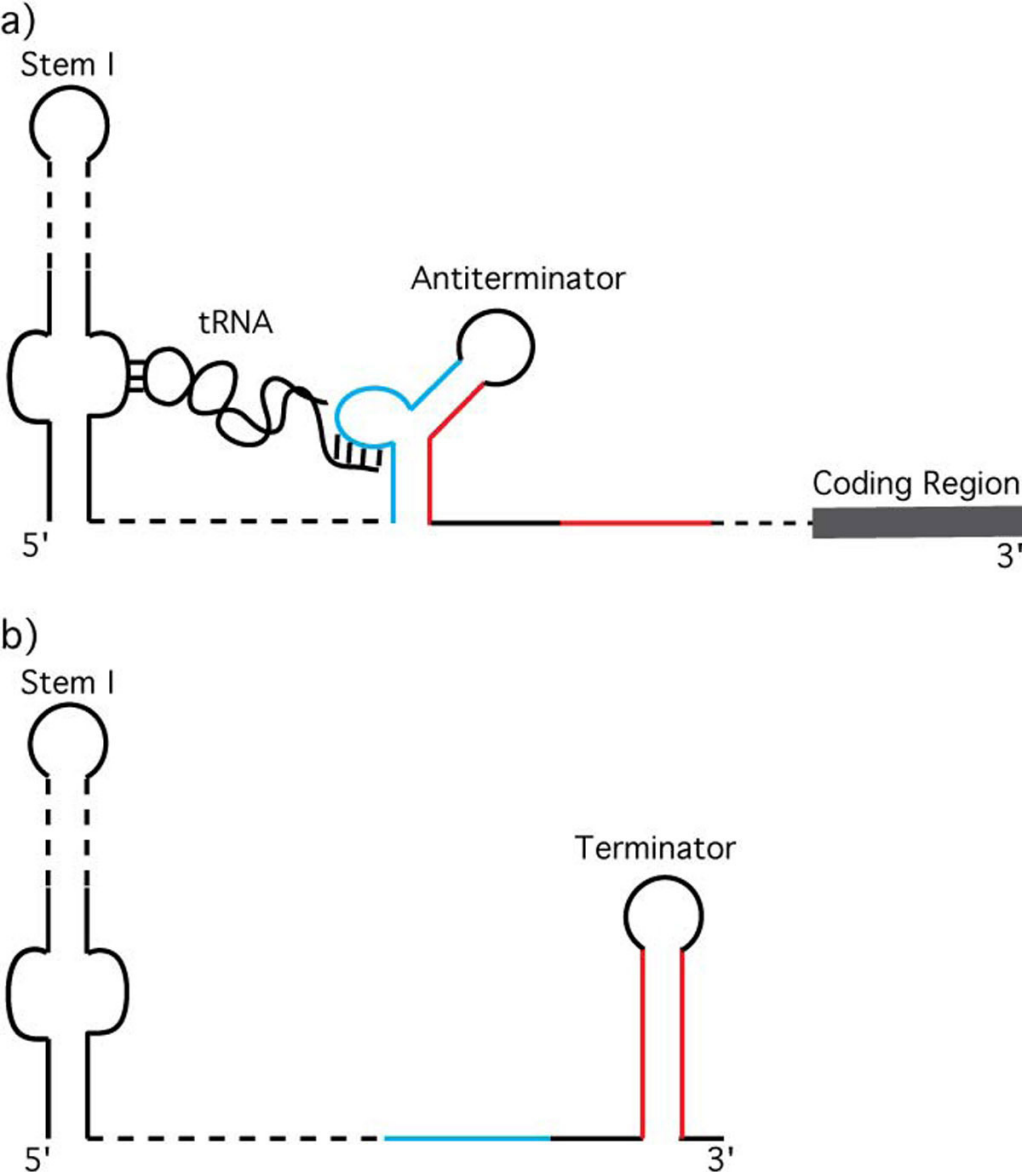
**T box transcription antitermination mechanism**. a) Uncharged, cognate tRNA binds the 5' -untranslated region of the nascent mRNA and stabilizes the antiterminator. b) In the absence of uncharged, cognate tRNA, the more stable terminator forms and transcription is terminated before the translation start site.

The T box riboswitch is an important regulatory mechanism found in Gram-positive bacteria including many pathogens [1-3]. The riboswitch responds to high levels of uncharged (non-aminoacylated) tRNA to control the transcription of cognate genes (e.g., aminoacyl tRNA synthetase, aaRS, genes) [3]. Cognate, uncharged tRNA binds the 5'-untranslated region of T box mRNA during transcription and, when present in sufficient quantities, results in antitermination (Figure 1a) [3]. The tRNA anticodon binds a specifier sequence in Stem 1, thus providing the cognate specificity, while the uncharged tRNA acceptor end nucleotides bind the first four bases in a seven nucleotide bulge of the highly conserved antiterminator structural element [4]. Aminoacylated-tRNA also binds the specifier sequence, but does not bind the antiterminator element [5]. In the absence of uncharged tRNA bound to the antiterminator element, a more thermodynamically stable stem-loop structure forms (the terminator) followed by factor-independent transcription termination a few nucleotides later [3]. The antiterminator and the terminator are mutually exclusive structural elements due to sharing common nucleotides (Figure 1b). Ligands that target and disrupt the T box riboswitch function could be potential antibacterial agents in light of the critical genes regulated by the T box mechanism [1,2].

We have been investigating the structure-function relationship of the T box antiterminator element and the key recognition features necessary for ligands to specifically bind the antiterminator and disrupt its function. There are few detailed medicinal chemistry studies of ligands targeting RNA [6] and this project has required an extensive multidisciplinary approach. The solution structure of antiterminator model RNA AM1A [7] was determined using molecular modelling with NMR-derived constraints [8]. The structure indicated that the seven-nucleotide bulge of the antiterminator was not pre-ordered for tRNA binding, but rather, that binding of the tRNA acceptor end must require a certain extent of tertiary-structure capture and/or an induced fit. Fluorescence life-time studies confirmed that modest antiterminator structural reorganization occurs upon tRNA binding in a magnesium-dependent manner [9]. While some RNAs have specific divalent metal ion binding sites, for the antiterminator RNA, the Mg^2+ ^binds via a diffuse, outer-sphere interaction [10]. In vitro selection studies of both the antiterminator [11] and tRNA [12] indicate that there are likely no direct interactions between the tRNA and the antiterminator other than the known base pairing between the acceptor end nucleotides and the first four nucleotides at the 5'-end of the seven-nucleotide bulge. Given this antiterminator structure-function information, ligands could potentially disrupt tRNA binding simply by competing with the base pairing between the tRNA acceptor end and the antiterminator bulge nucleotides.

Aminoglycosides bind AM1A in a structure-specific manner, most likely via electrostatics [13,14]. In contrast, two novel classes of heterocyclic compounds, triazoles and oxazolidinones, bind AM1A with structure-specificity and high affinity, but without reliance on electrostatics [15-18] and can alter T box transcription antitermination [16]. A computational, quantitative structure activity relationship analysis has shown that hydrophobic interactions play a significant role in the binding of these compounds to AM1A [19]. Furthermore, the small molecule ligands disrupt tRNA binding to the antiterminator in a structure-specific manner [20].

**Figure 2:**
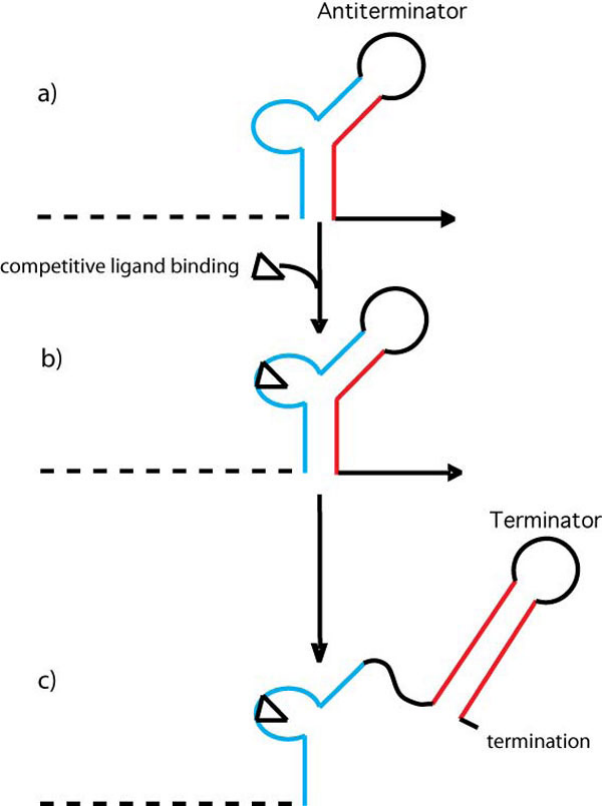
**T box drug discovery goal**. As soon as the antiterminator is transcribed (a), a small molecule ligand competes with tRNA for binding the antiterminator (b), but does not overly stabilize the antiterminator such that the terminator can form and transcription is terminated (c).

From a drug discovery perspective, a key factor to determine is the range of ligand-induced stabilization that can be accommodated without overly stabilizing the antiterminator element and precluding terminator formation. The goal of our T box drug discovery project is to determine the key ligand features that lead to specific antiterminator binding, but that do not result in excessive stabilization of the antiterminator secondary structure. These ligands could then potentially compete with tRNA for binding to the antiterminator, but still allow terminator formation such that transcription of a T box gene critical for bacterial survival would be terminated (Figure 2). The predicted thermodynamic stability (ΔG) of the terminator and antiterminator structural elements have been compared for the *B. subtilis tyrS *T box [4]. However, there has been no systematic comparison of predicted thermodynamic stability differences for a larger set of T box genes. Using a structural bioinformatics approach, we have analyzed the differences in predicted free energy (ΔΔG) between antiterminators and terminators in a set of aaRS T box genes in order to predict an upper limit of ligand-induced stabilization that can potentially be accommodated.

## Methods

The thermodynamic stability of T box antiterminator and terminator structural elements was calculated using the DINAMelt server [21]. The DINAMelt server computes the secondary structure and free energy of the folded RNA using a secondary structure folding algorithm and empirically-derived nearest neighbour coefficients [21]. The folding algorithm predicts the minimum energy RNA secondary structure using the available thermodynamic data for base pairing, base stacking and destabilizing energies [22,23]. The sequences analyzed were predicted to be involved in aaRS T box antiterminator and terminator structural elements from *Bacillus cereus *(BC), *Bacillus subtilis *(BS), *Clostridium botulinum *(CB), *Clostridium difficile *(CDF), *Clostridium perfringens *(CPE), *Staphylococcus aureus *Mu50 (SA), *Streptococcus agalactiae *(SAG), and, *Streptococcus pyogenes *(SPY) [2]. The ΔG for each aaRS T box antiterminator and terminator sequence was determined using the Quickfold (RNA 3.0) option on the DINAMelt server and the predicted thermodynamic stability difference calculated from ΔΔG = ΔG_Terminator _- ΔG_Antiterminator_. The % suboptimal setting was adjusted as necessary to obtain the lowest free energy antiterminator fold that had the consensus secondary structure of the core seven-nucleotide bulge containing the 5' -UGGN-3' nucleotides that are complementary to the tRNA acceptor end nucleotides [4].

## Results and discussion

**Figure 3:**
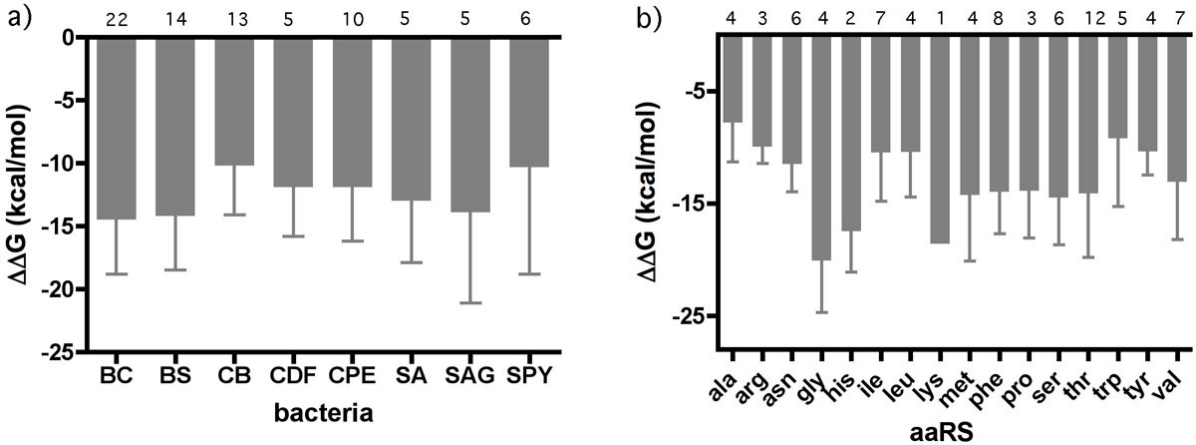
**Average calculated **ΔΔ**G**. Average calculated aaRS ΔΔG values grouped by a) bacteria and b) aaRS. Standard deviation indicated by bar and number of aaRS sequences averaged noted over the individual columns.

The free energy values for the antiterminator and terminator structural elements of the T box genes analyzed were calculated using the DINAMelt webserver [21]. The predicted ΔG values are listed in Additional File 1 and the relative comparisons of terminator vs.antiterminator stability (ΔΔG) are summarized in Figure 3. The overall average ΔΔG for all aaRS studied was -12.8 kcal/mol. The average ΔΔG values did not differ significantly between bacteria when comparing between the pathogenic bacteria nor between pathogenic vs. the non-pathogenic bacteria studied (BS) (Figure 3a). In contrast, there were significant differences in ΔΔG averages between specific aaRS with alanyl aaRS having the smallest average ΔG (-7.8 ± 3.5 kcal/mol) and glycyl aaRS having the largest (-20.1 ± 4.6 kcal/mol) (Figure 3b). Based on these results, the glycyl aaRS, on average, may be best able to accommodate ligand-induced stabilization of the antiterminator and still allow formation of the terminator. An important factor to consider, however, is that the free energy calculations are based on empirically-derived parameters for known RNA structural motifs [21]. Structural motifs, especially in loops and bulges, that have not been previously characterized might contribute to the stability of the RNA elements and not be accounted for in the DINAMelt ΔG calculations. Since the loop of the antiterminator is not highly conserved [4], most likely there is no structural motif within it that might contribute to the antiterminator stability, however, the possibility cannot be excluded. Further investigation of experimentally-derived free energy values of individual antiterminators and terminators is needed.

## Conclusions

The free energy of T box riboswitch antiterminator and terminator elements was predicted and compared for a series of aaRS T box genes. The observed aaRS-specific stability differences between these key riboswitch structural elements could potentially be targeted to effect ligand-specificity in future drug discovery efforts.

## Abbreviations

(aaRS): Aminoacyl tRNA synthetase; (BC): *Bacillus cereus*; (BS): *Bacillus subtilis*; (CB): *Clostridium botulinum*; (CDF): *Clostridium difficile*; (CPE): *Clostridium perfringens*; Mu50 (SA): *Staphylococcus aureus*; (SAG): *Streptococcus agalactiae *and, *Streptococcus pyogenes *(SPY).

## Competing interests

JH is a co-inventor on U.S. Patent number 7,005,441 for T box binding assays and ligands.

## Authors' contributions

FJ carried out the sequence folding and free energy predictions. JH conceived of the study, designed and coordinated the study and prepared the manuscript.

## Supplementary Material

Additional file 1 **Predicted ΔG values for aaRS T box terminators and antiterminators**. Click here for file
