# Supplementary material for: Interfacing medicinal chemistry with structural bioinformatics: implications for T box riboswitch RNA drug discovery
Source: BMC Bioinformatics. 2012 Mar 13;13(Suppl 2):S5. doi: 10.1186/1471-2105-13-S2-S5 (PMC3375634; doi:10.1186/1471-2105-13-S2-S5)
Supplement: Additional file 1 — Predicted ΔG values for aaRS T box terminators and antiterminators. [file 1471-2105-13-S2-S5-S1.pdf]

Interfacing medicinal chemistry with structural bioinformatics: Implications for T box riboswitch RNA drug discovery

Franziska Jentzsch, Jennifer V. Hines

Additional File 1

Table 1. Predicted ΔG values for aaRS T box terminators and antiterminators

Aminoacyl-tRNA synthetase gene[a]	ΔG Terminator[b] (kcal/mol)	ΔG Antiterminator[b] (kcal/mol)	ΔΔG[c] (kcal/mol)
BS_ALAS	-18	-12.1	-5.9
BC_ALAS	-21.6	-8.6	-13
SA_ALAS	-13.1	-6.9	-6.2
CB_ALAS	-12.7	-6.6	-6.1
BC_ARGS2	-17.1	-6.1	-11
CB_ARGS	-16.4	-5.7	-10.7
CPE_ARGS	-14.5	-6.3	-8.2
BC_ASNS2	-16.1	-4.7	-11.4
BC_ASNS3	-21	-6.8	-14.2
CB_ASNS2	-19.3	-4.8	-14.5
CB_ASNS3	-11.8	-1.4	-10.4
CDF_ASNS2	-21.1	-10	-11.1
CPE_ASNS2	-15.3	-7.4	-7.9
BS_GLYQ	-29.5	-10.9	-18.6
BC_GLYS2	-29.4	-14.8	-14.6
SAG_GLYQ	-34.7	-9.3	-25.4
SPY_GLYQ	-30.3	-8.6	-21.7
BS_HISS	-28.2	-8.2	-20
BC_HISS	-22.6	-7.7	-14.9
BS_ILES	-23.1	-11	-12.1
BC_ILES	-27.8	-8.4	-19.4
BC_ILES2	-21.6	-12.2	-9.4
CB_ILES	-12.6	-5.3	-7.3
CPE_ILES	-12.1	-2.9	-9.2
SAG_ILES	-11.7	-2	-9.7
SPY_ILES	-8.7	-2	-6.7
BS_LEUS	-20.4	-13.3	-7.1
BC_LEUS	-24	-8	-16
CB_LEUS	-13.7	-5.7	-8
CPE_LEUS	-17.8	-7.1	-10.7
BC_LYSS	-24.7	-6.1	-18.6
BC_METS	-31.1	-8.3	-22.8
CB_METS	-17.1	-3.8	-13.3
CDF_METS	-16.5	-6.3	-10.2
CPE_METS	-17.5	-6.8	-10.7
BS_PHES	-18.4	-4.7	-13.7
BC_PHES	-15.7	-5	-10.7

SA_PHES	-23.2	-4.7	-18.5
CB_PHES	-14.8	-6	-8.8
CDF_PHES	-17.6	-6.8	-10.8
CPE_PHES	-22.1	-5.9	-16.2
SAG_PHES	-20.9	-6.8	-14.1
SPY_PHES	-25.7	-6.8	-18.9
BC_PROS	-20.5	-8.5	-12
CDF_PROS	-24.6	-5.8	-18.8
CPE_PROS	-16.7	-5.7	-11
BS_SERS	-26.7	-9	-17.7
BC_SERS	-24.8	-9.5	-15.3
SA_SERS	-21.7	-4.9	-16.8
CB_SERS2	-21.9	-5	-16.9
CB_SERS1	-15.6	-9.3	-6.3
CPE_SERS	-17.4	-3.3	-14.1
BS_THRS	-20.4	-4.6	-15.8
BS_THRZ	-21.3	-5.6	-15.7
BS_THRZ*	-21.1	-7.3	-13.8
BC_THRS	-18.7	-5.1	-13.6
BC_THRZ	-25.5	-7.1	-18.4
BC_THRZ*	-29.4	-6.9	-22.5
SA_THRS	-16.9	-4.7	-12.2
CB_THRZ	-16.5	-5.1	-11.4
CDF_THRZ	-14.4	-5.7	-8.7
CPE_THRS	-28.7	-7	-21.7
SAG_THRS	-17.9	-3.6	-14.3
SPY_THRS	-6.3	-5.1	-1.2
BS_TRPS	-20.6	-4.7	-15.9
BC_TRPS	-20.4	-5.7	-14.7
BC_TRPS2	-15.1	-6	-9.1
CB_TRPS	-11.9	-7.7	-4.2
SPY_TRPS	-11.9	-9.6	-2.3
BS_TYRS	-18.8	-6.4	-12.4
BS_TYRZ	-18.9	-7.8	-11.1
BC_TYRS	-16.7	-6.1	-10.6
BC_TYRS2	-12.2	-4.8	-7.4
BS_VALS	-24.4	-5.1	-19.3
BC_VALS	-23.3	-3.8	-19.5
SA_VALS	-18	-6.6	-11.4
CB_Y5-VALS	-19.8	-4.8	-15
CPE_VALS	-13.1	-4.1	-9
SAG_Y1VALS	-13.7	-7.5	-6.2
SPY_Y1VALS	-20.6	-9.6	-11

[a]Abbreviation as defined in reference [2].

[b]ΔG values determined using DINAMelt (Quickfold RNA 3.0), see Methods for details.

[c]$\Delta\Delta G = \Delta G_{terminator} - \Delta G_{antiterminator}$
